# Savannah Medical College

**Published:** 1873-07

**Authors:** 


### SAVANNAH MEDICAL COLLEGE.

We take pleasure in calling attention to the annual announcement of the Savannah Medical College, for the session of 1873–'74. It is manifest that the Faculty are earnestly at work in the effort to uphold a high standard of medical education, and with the clinical advantages which they are furnishing and the decided ability which they undoubtedly possess to make their facilities available, it is our sincere hope that the success of the institution may fully reward their labors.

We are pleased to notice that they have not followed the bad example furnished by a number of the medical colleges of the country in lowering the old standard of fees. The cheapening of medical education can not possibly accomplish any good, and as a medical student we should select (other things being equal) an institution that had not fallen into this policy of apparently bidding for students from a low stand-point.

The introductory lecture of the course will be delivered by Prof. W. G. Bulloch, M.D., on Monday, November 3, 1873. Among other advantages enumerated by the Faculty, we call attention, as worthy of notice, to the following: "The climate of Savannah presents inducements second to none in the country. Students from the Northern, Northwestern, and Western States, who, in selecting a medical school of high standing, desire also to combine with it geniality of climate, and also to avoid the rigor and unhealthfulness of a more northern latitude, will find such an opportunity presented by the Savannah Medical College. The proximity of the city of Savannah to Florida, and the acknowledged benefit of this climate upon invalids and persons predisposed to pulmonary diseases, are attested annually by the thousands who seek this city as a summer resort."

---

Special Notice.—*The Editors have no connection with the business affairs of the* Journal, *and can not attend even to correspondence of that character.*

